# Acting locally - affecting globally: RNA sequencing of gilthead sea bream with a mild *Sparicotyle chrysophrii* infection reveals effects on apoptosis, immune and hypoxia related genes

**DOI:** 10.1186/s12864-019-5581-9

**Published:** 2019-03-11

**Authors:** M. Carla Piazzon, Ivona Mladineo, Fernando Naya-Català, Ron P. Dirks, Susanne Jong-Raadsen, Anamarija Vrbatović, Jerko Hrabar, Jaume Pérez-Sánchez, Ariadna Sitjà-Bobadilla

**Affiliations:** 1 Fish Pathology Group, Institute of Aquaculture Torre de la Sal (IATS-CSIC), Ribera de Cabanes, Castellón, Spain; 2 Institute of Oceanography and Fisheries, Split, Croatia; 3 Nutrigenomics and Fish Growth Endocrinology Group, Institute of Aquaculture Torre de la Sal (IATS-CSIC), Ribera de Cabanes, Castellón, Spain; 4 Biotechvana, Parc Cientific, Universitat de Valencia, Valencia, Spain; 5 Future Genomics Technology, Leiden, The Netherlands

**Keywords:** *Sparus aurata*, *Sparicotyle chrysophrii*, Gills, Monogenea, Ectoparasites, Illumina RNA-seq, Transcriptomics, Apoptosis, Immune response

## Abstract

**Background:**

Monogenean flatworms are the main fish ectoparasites inflicting serious economic losses in aquaculture. The polyopisthocotylean *Sparicotyle chrysophrii* parasitizes the gills of gilthead sea bream (GSB, *Sparus aurata*) causing anaemia, lamellae fusion and sloughing of epithelial cells, with the consequent hypoxia, emaciation, lethargy and mortality. Currently no preventive or curative measures against this disease exist and therefore information on the host-parasite interaction is crucial to find mitigation solutions for sparicotylosis. The knowledge about gene regulation in monogenean-host models mostly comes from freshwater monopysthocotyleans and almost nothing is known about polyopisthocotyleans. The current study aims to decipher the host response at local (gills) and systemic (spleen, liver) levels in farmed GSB with a mild natural *S. chrysophrii* infection by transcriptomic analysis.

**Results:**

Using Illumina RNA sequencing and transcriptomic analysis, a total of 2581 differentially expressed transcripts were identified in infected fish when compared to uninfected controls. Gill tissues in contact with the parasite (P gills) displayed regulation of fewer genes (700) than gill portions not in contact with the parasite (NP gills) (1235), most likely due to a local silencing effect of the parasite. The systemic reaction in the spleen was much higher than that at the parasite attachment site (local) (1240), and higher than in liver (334). NP gills displayed a strong enrichment of genes mainly related to immune response and apoptosis. Processes such as apoptosis, inflammation and cell proliferation dominated gills, whereas inhibition of apoptosis, autophagy, platelet activation, signalling and aggregation, and inflammasome were observed in spleen. Proteasome markers were increased in all tissues, whereas hypoxia-related genes were down-regulated in gills and spleen.

**Conclusions:**

Contrasting forces seem to be acting at local and systemic levels. The splenic down-regulation could be part of a hypometabolic response, to counteract the hypoxia induced by the parasite damage to the gills and to concentrate the energy on defence and repair responses. Alternatively, it can be also interpreted as the often observed action of helminths to modify host immunity in its own interest. These results provide the first toolkit for future studies towards understanding and management of this parasitosis.

**Electronic supplementary material:**

The online version of this article (10.1186/s12864-019-5581-9) contains supplementary material, which is available to authorized users.

## Background

Vegetable and animal production face the challenge to feed the rapidly growing human population, and aquaculture is expected to be a major provider, with a constant increase in volume and number of farmed species in the last two decades. In fact, in 2015 world aquaculture already surpassed wild fisheries, reaching 53.1% [1], and this contribution is projected to reach 62% by 2030 [2]. In the Mediterranean region, gilthead sea bream (GSB, *Sparus aurata*) is one of the main cultured species, with an uneven production among countries, some of them having impressive growth rates and others having stagnated or decreased trends [3]. The only way to increase productivity is by addressing the prophylaxis and mitigation of several diseases, as the impact of pathogens on aquaculture is substantial; the financial losses are estimated to be roughly 20% of the total production value [4]. Among them, it is estimated that the world annual grow-out loss due to parasites in finfish farming ranges from 1 to 10% of harvest size, with an annual cost of $1.05 to $9.58 billion [5]. These economic losses incurred from parasitic diseases are due not exclusively to direct mortalities, but also to decreases in growth performance, feed conversion and product quality, low reproduction efficacy, increased susceptibility to other diseases and negative public image of aquaculture in a long term [5, 6]. The production of GSB is hampered by recurrent, seasonal proliferations of the ectoparasite *Sparicotyle chrysophrii* (syn. *Microcotyle chrysophrii*) (Monogenea: Polyopisthocotylea), parasitizing the gill epithelium. It has been suggested that this monogenean causes an increase by > 0.4 of the total feed conversion rate (FCR) of GSB, which translates in an increased feed requirement for over 50,000 tons along production (Rigos G., unpublished. data).

Sparicotylosis clinical signs include lethargy due to hypoxia and severe anaemia [7], histopathologically reflected in lamellar shortening, clubbing and synechiae, proliferation of the epithelial tissue, resulting in fusion of the secondary lamellae, and abundant chloride cells [8]. Severe pathogenicity including anaemia, lamellae fusion and sloughing of epithelial cells can be detected even at moderate intensity of infection levels, with eight parasites per gill arch [9]. However, low infection intensity values can already significantly decrease some haematological values such as haemoglobin (Hb). In fact, in experimentally infected small GSB harbouring an average of 2.2 monogeneans/fish, Hb was significantly decreased [8]. Changes in gills are followed by a dramatic increase in size and number of splenic melanomacrophage centres [10], most likely in an effort of the host to mitigate the increased hemosiderin and lipofuscin metabolism caused by erythrocyte destruction, tissue catabolism or collateral degenerative chronic disorder [11, 12]. Secondary and concomitant infections with other parasites and bacteria in *S. chrysophrii*-infected GSB are common [13–15].

The information regarding the host immune response to *Sparicotyle*, beyond histopathology, is very recent. The parasite infection seems to trigger a cellular response of the fish immune system, while it inhibits its humoral response. Parasitized fish attempt to control the parasite infection through the action of reactive oxygen species, but they may become more sensitive to secondary bacterial or other parasitic infections [16]. This phenomenon was demonstrated not only through significant differences in innate immune factors between infected and control fish, but also through strong correlations between some of those factors and parasite load, fish weight and/or Hb concentration [16]. The knowledge about monogenean-host immune modulation comes mainly from freshwater monogeneans of the genus *Gyrodactylus* infecting salmonids and sticklebacks [17]. Unlike in *Sparicotyle*-GSB interaction, the number of *Gyrodactylus* parasites tends to decline 20–30 days post-infection, suggesting an adaptive immune mechanism. The first studies showed that macrophage secretion of complement factor 3 (C3) and interleukin 1β (Il1β) induced epithelial hyperplasia and mucus secretion [18], and later on, the picture of the immune response to monogeneans became more complex, and differed depending on the host and the specific parasite studied [19–27]. To our knowledge, there is no information about the genes involved in GSB response or comprehensive studies on the fish host response to monogenean infection inferred by massive sequencing.

Currently there are no preventive or curative measures against this disease and the only treatment available for the control of *S. chrysophrii* are formalin baths. However, the use of formalin in open seawater, poses multiple environmental and workplace safety concerns in addition to large operational costs [28]. Furthermore, formalin is quite toxic for fish and sea life, and the margin between parasiticidal effectivity and induction of severe negative effects is quite narrow [28]. As a consequence, use of formalin in open seawater is already banned in some countries and it will probably be banned in Europe in the future [29]. There are no other registered pharmaceutical products for the control of *S. chrysophrii* and therefore there is an urgent need to find solutions for this parasitosis. Thus, understanding the host response elicited by the parasite would help to find solutions to manage this infection. The current study aims to set bases for deciphering host response at local (gills) and systemic (spleen, liver) levels in farmed GSB with a mild natural *S. chrysophrii* infection by transcriptomic analysis, by defining the most relevant pathways involved in the pathology.

## Results

### *Sparicotyle chrysophrii* induces local and systemic effects

A preliminary PCR-array, profiling the expression levels in gills and spleen of 45 selected genes related to hypoxia, inflammation, iron metabolism, tight junction proteins, immune system, mucins, apoptosis, antioxidant activity and cell growth and regeneration showed that *Sparicotyle* infections had a potent effect both locally (gills) and systemically (spleen) (Additional file 1A). Interestingly, the tissues showing more changes when compared to control uninfected fish were the spleen and the portions of the gills where the parasite was not present (NP, non-parasitized gills). The gill portions with attached parasites (P, parasitized gills) showed fewer significant changes. The hypoxia-related gene *hif1α* showed a strong down-regulation in the spleen and both gill portions. Several cytokines and lymphocyte markers were feebly up-regulated, mainly in the spleen. Genes involved in cell growth and tissue regeneration were up-regulated in the gills, both P and NP. This preliminary profiling allowed selecting the animals with higher quality samples to be subjected to RNA sequencing.

**Fig. 1:**
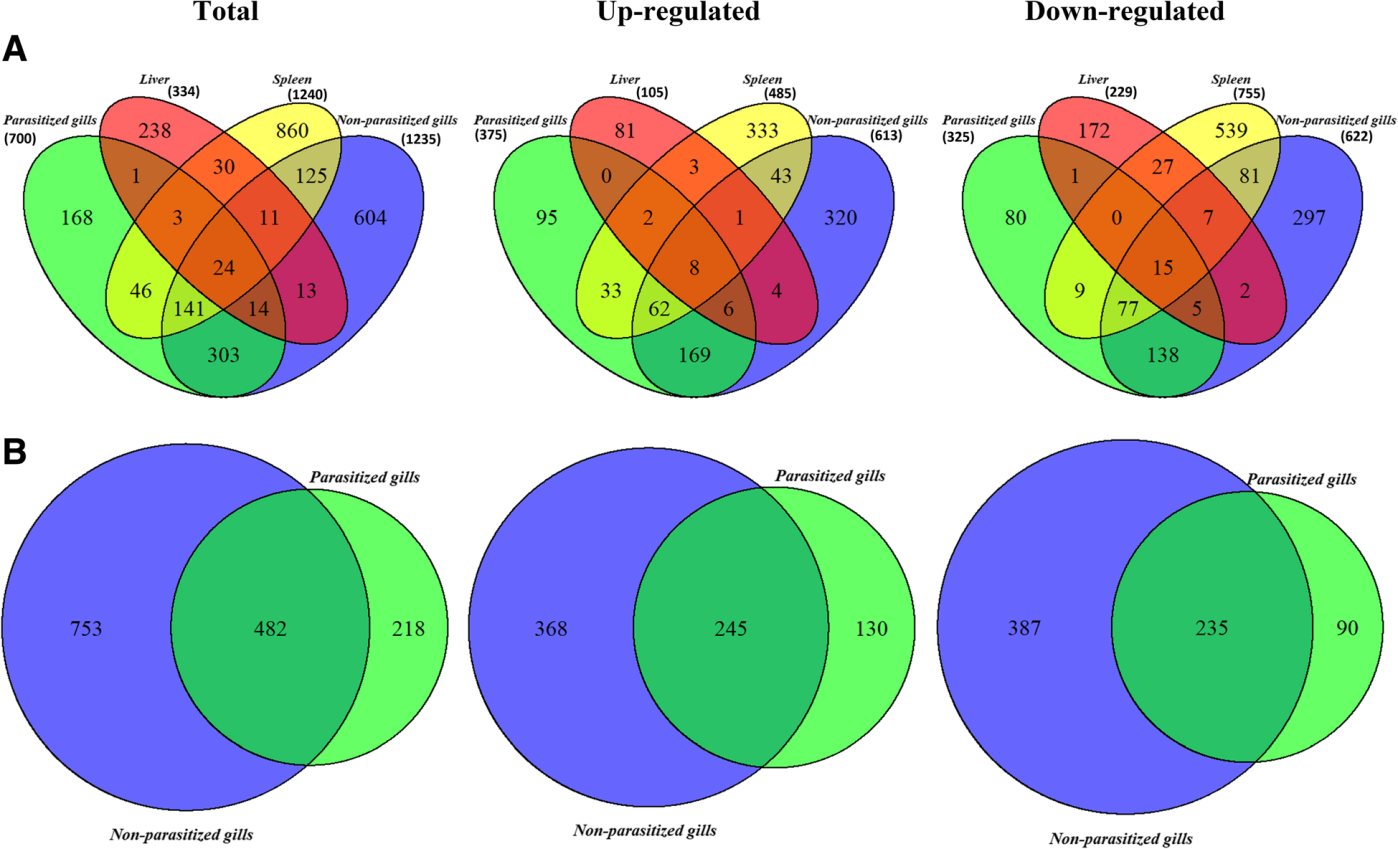
Venn diagrams showing shared and unique differentially regulated transcripts among all tissues (**a**) and between parasitized (P) and non-parasitized (NP) gill portions (**b**). Venn diagrams on the left (Total) represent all differentially expressed transcripts of infected fish when compared to control uninfected animals, whereas the middle and right ones represent separately up- and down-regulated transcripts respectively. Numbers in parenthesis (**a**) indicate the number of differentially expressed transcripts in each tissue

In the RNA sequencing analysis, a total of 2581 differentially expressed (DE) transcripts were identified among all the studied tissues when comparing infected (INF) and control (CTRL) groups. The proportion of total up- or down-regulated transcripts among tissues was quite balanced, having 55.5% of transcripts down-regulated and 45.5% up-regulated. Figure 1A shows the number of differentially expressed transcripts per tissue. The spleen and NP gills showed the highest numbers of DE transcripts (1240 and 1235, respectively) and the liver the lowest (334). The proportion of up- and down-regulated transcripts in the gills was approximately 50%, whereas down-regulated genes prevailed in the spleen and the liver (60.9 and 68.6%, respectively).

The numbers of shared and unique total, up- and down-regulated DE transcripts among tissues is shown in Fig. 1A. When comparing all tissues, 8 transcripts were found to be up-regulated in all tissues and 15 were commonly down-regulated. As expected, the tissues that showed more common DE transcripts were the gills (P and NP). The liver was the tissue with fewer shared DE genes. When comparing P and NP gills (Fig. 1B), a higher number of DE genes were found when the parasite was not present (NP), in agreement with the preliminary PCR-array results. Out of the 1453 DE transcripts in both gill tissues, only 33.2% (482) had shared regulation, while 51.8% (753) and 15% (218) were specifically regulated in NP and P gills, respectively.

### The gills and the spleen are the most responsive organs to *Sparicotyle chrysophrii* infections

**Fig. 2:**
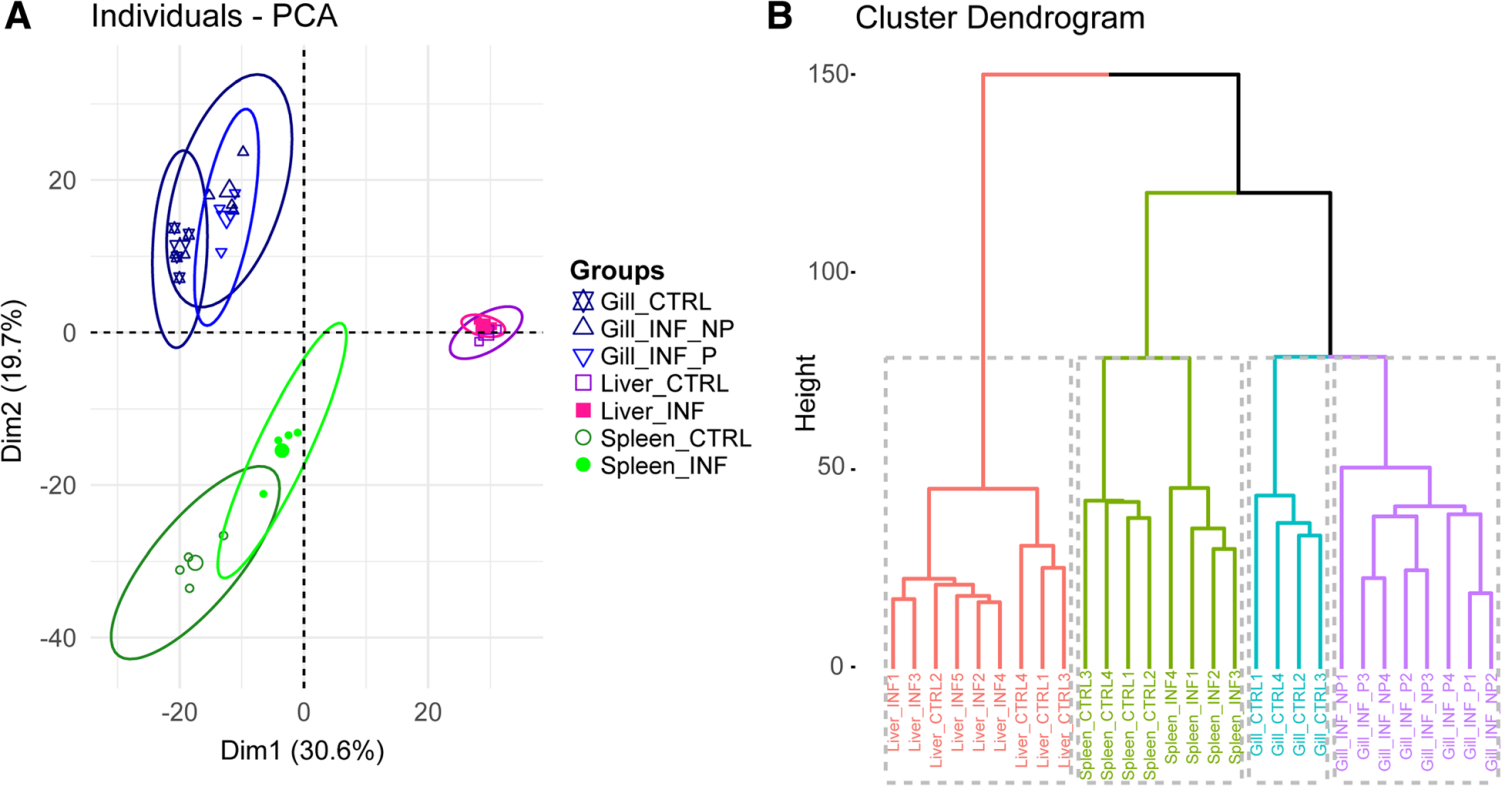
Principal component analysis (**a**) and hierarchical clustering (**b**) of all the individual samples constructed based on the FPKM values for each of the differentially regulated transcripts. The optimal number of clusters in (**b**) was determined by the gap statistic (4). CTRL stands for control uninfected and INF refers to parasite infected fish. INF gill samples were separated in parasitized (P) and non-parasitized (NP) gill sections depending on the presence or absence of the parasite in that particular area

Principal component analysis (PCA) of the expression of all DE transcripts in all studied tissues showed a clear clustering by organ type, which is expected due to the specific expression pattern per tissue. Infection with the parasite induced a shift in infected gills (both P and NP) and the spleen, whereas changes were not very prominent in the liver (Fig. 2A). The optimal number of clusters determined by the gap statistic was 4. Cluster analysis separated the different tissues and clustered in a separate group the gills from control uninfected animals, evidencing that the most significant effects were taking place in the target tissue (Fig. 2B). Cluster analysis clearly separated in different branches CTRL (control) and INF (infected) groups in the spleen. However, the clustering in the liver was not that distinct, showing a feeble effect of the infection on this organ.

### Functional gene enrichment analysis

Out of the 2581 differentially expressed transcripts among all tissues, 1710 (66.25%) were identified as known protein-coding and successfully annotated, including gene ontology (GO) term assignment. These known genes were further used to obtain information about the biological implications of the parasite-induced changes. GO enrichment analysis based on the number of DE genes per tissue revealed a large number of enriched GO terms (FDR cutoff 0.05), shown in Table 1. A detailed list of the GO enrichment analysis and results is shown in Additional file 2.

**Table 1:** Gene ontology terms significantly enriched in the different tissues

Tissue	GO Level	Total	Up-regulated	Down-regulated
Liver	BP	101	0	99
	CC	12	0	10
	MF	26	0	27
	Total	139	0	136
Spleen	BP	192	22	159
	CC	37	3	35
	MF	48	12	35
	Total	277	37	229
Parasitized (P) gills	BP	41	3	7
	CC	12	7	5
	MF	11	5	2
	Total	64	15	14
Non-parasitized (NP) gills	BP	184	75	87
	CC	33	16	23
	MF	51	28	31
	Total	268	119	141

GO term enrichment (FDR cutoff 0.05) was calculated using all differentially expressed genes (Total, first column) or separating up- and down-regulated (second and third column respectively) genes per tissue. BP: biological process; CC: cellular component; MF: molecular function

**Fig. 3:**
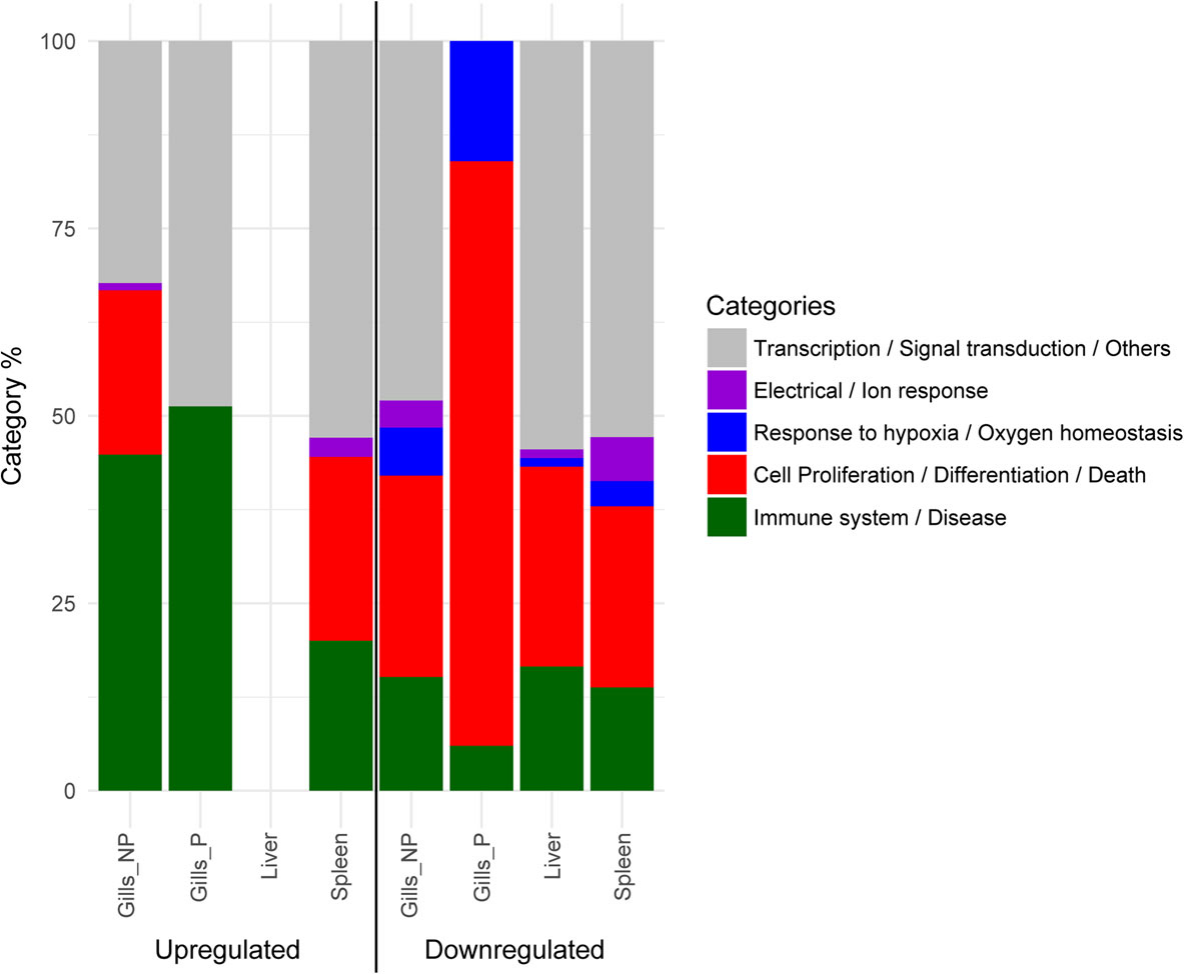
Percentage of broad gene ontology (GO) categories up- or down-regulated in the different tissues. Significantly enriched GO terms up- (left) and down-regulated (right) in each infected tissue relative to the uninfected control were grouped in five broad categories and their abundance is represented as percentage among all significantly enriched terms. Gill samples were separated in parasitized (P) and non-parasitized (NP) gill sections

To facilitate the interpretation of the results, GO categories were grouped in five key broad functional categories based on GO term characterization and the specific pathology and organs studied. The chosen categories were: 1) Immune system and disease; 2) Cell proliferation, differentiation and death; 3) Response to hypoxia and oxygen homeostasis; 4) Response to ions or electrical stimulus; and 5) Transcription, signal transduction and others (incl. metabolism). It is interesting to note that the category “Response to hypoxia and oxygen homeostasis” was always down-regulated, being highly present in the gills, both P and NP. The category “Cell proliferation, differentiation and death” was highly represented among down-regulated genes of parasitized gills. “Immune system and disease” was more abundant among the up-regulated categories in P and NP gills (Fig. 3).

### Pathway analysis

**Fig. 4:**
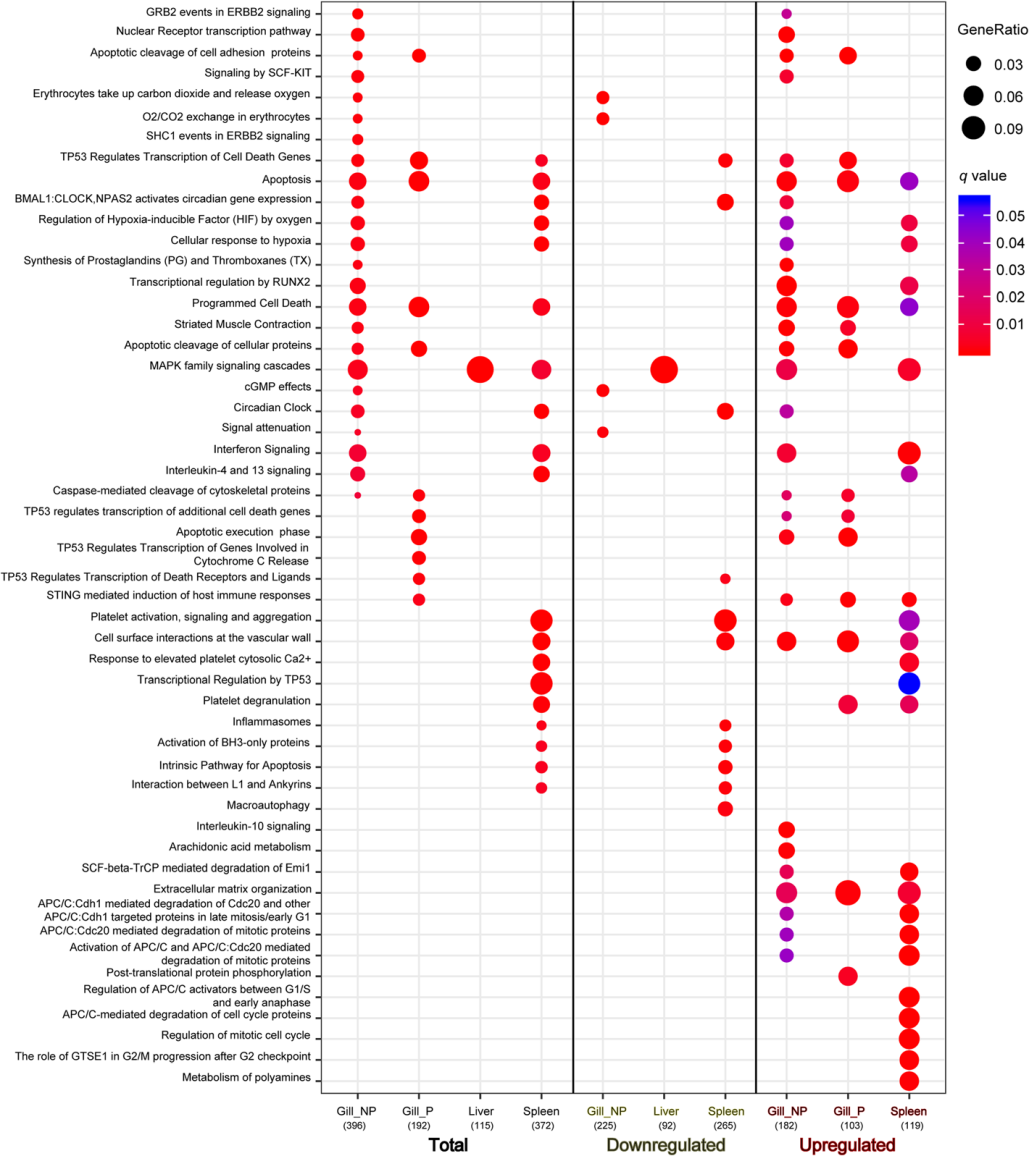
Dotplot pathway enrichment map showing the significantly overrepresented pathways (*q* < 0.05) when considering all differentially regulated genes (Total, left) or the down-regulated (middle) or up-regulated (right) set of genes in non-parasitized (NP) gills, parasitized (P) gills, liver and spleen of infected animals when compared to the uninfected controls

DE genes were classified in broad pathways using the Reactome database for biological pathways (https://reactome.org). The fact that pathway analysis databases are constructed for model species, mainly for human, implied that only 60% of our identified sequences could be successfully transformed in a known format for the pathway analysis software. Despite these limitations, an interesting overview could be extracted from the pathway analysis (Additional file 3 and Fig. 4). Metabolism and signal transduction were the pathways where the majority of the DE proteins mapped, followed by immune system, which coincided with the higher representation of these pathways in the Reactome database (Additional file 3). The relative percentage of genes related to immune system, programmed cell death and developmental biology was higher in P gills, coinciding with what was observed in the GO analysis in Fig. 3. Interestingly, the marked pathway of extracellular matrix organisation was present in both P and NP gills. In the latter, programmed cell death was also very prominent compared to other tissues, while DNA repair and replication was the least represented. In the liver and the spleen, genes involved in cellular response to external stimuli and cell cycle appeared in a higher percentage when compared to the other tissues.

To assess statistically significant representations, pathway analysis was performed using the set of genes that were significantly up- or down-regulated (*q* value < 0.05) per tissue (Fig. 4). Non-parasitized gills showed significant down-regulation of O_2_/CO_2_ exchange pathways with up-regulation of apoptosis and immune system pathways. In parasitized gills, no pathway was significantly represented among the down-regulated genes, but amongst the up-regulated pathways, apoptosis was dominantly represented. In the spleen, cell proliferation was represented among the up-regulated genes, together with interferon signalling. In the liver, only significant down-regulation of MAPK signalling cascades was found, with no overrepresented pathway amongst the up-regulated genes. This comparison evidences once again that the most numerous changes were occurring in NP gills, followed by the spleen, and the liver was the least affected tissue.

### Relevant differentially expressed genes

**Fig. 5:**
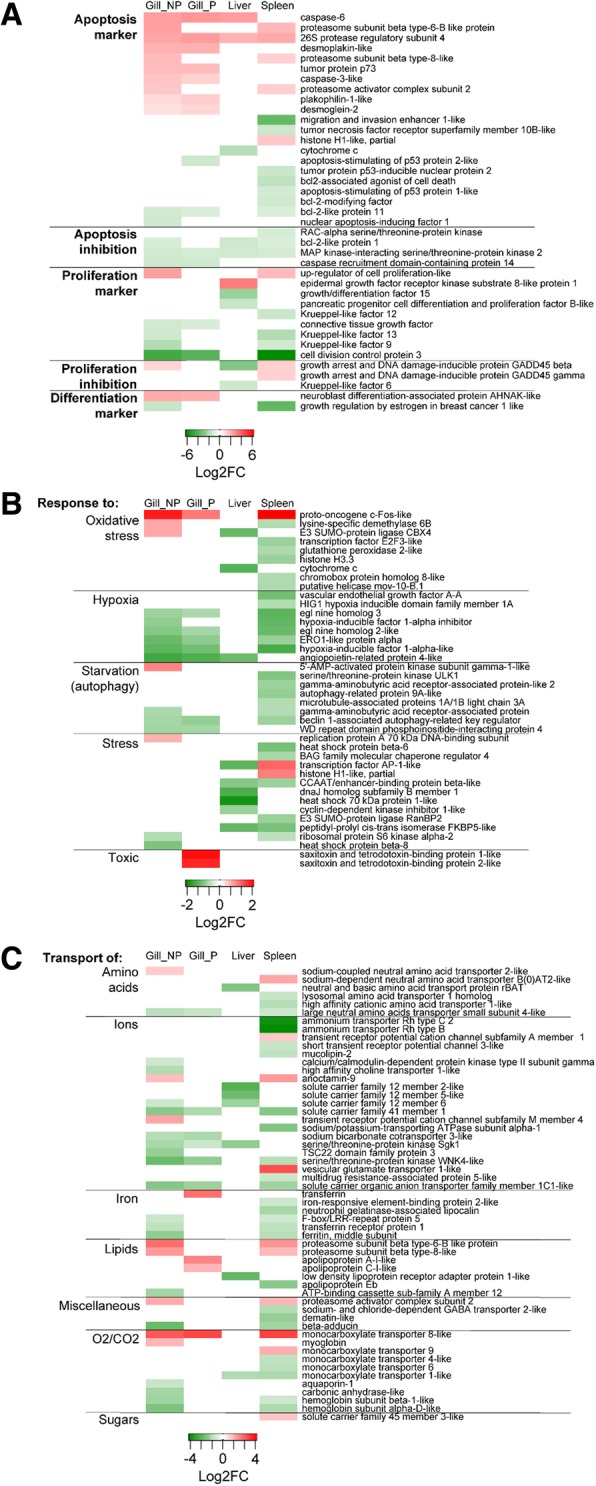
Heatmaps depicting the log2 fold change (FC) of the selected genes involved in Cell death and proliferation (**a**), Cellular response to external stimuli (**b**) and Transport of small molecules (**c**). Genes that were not differentially expressed between the infected and the control uninfected groups appear white. Genes that were significantly up- or down-regulated (*q* value < 0.05) by *Sparicotyle chrysophrii* infection appear in shades of red or green (respectively). To facilitate the interpretation of the results, genes were grouped in different subcategories. Gill samples were separated in parasitized (P) and non-parasitized (NP) gill sections

**Fig. 6:**
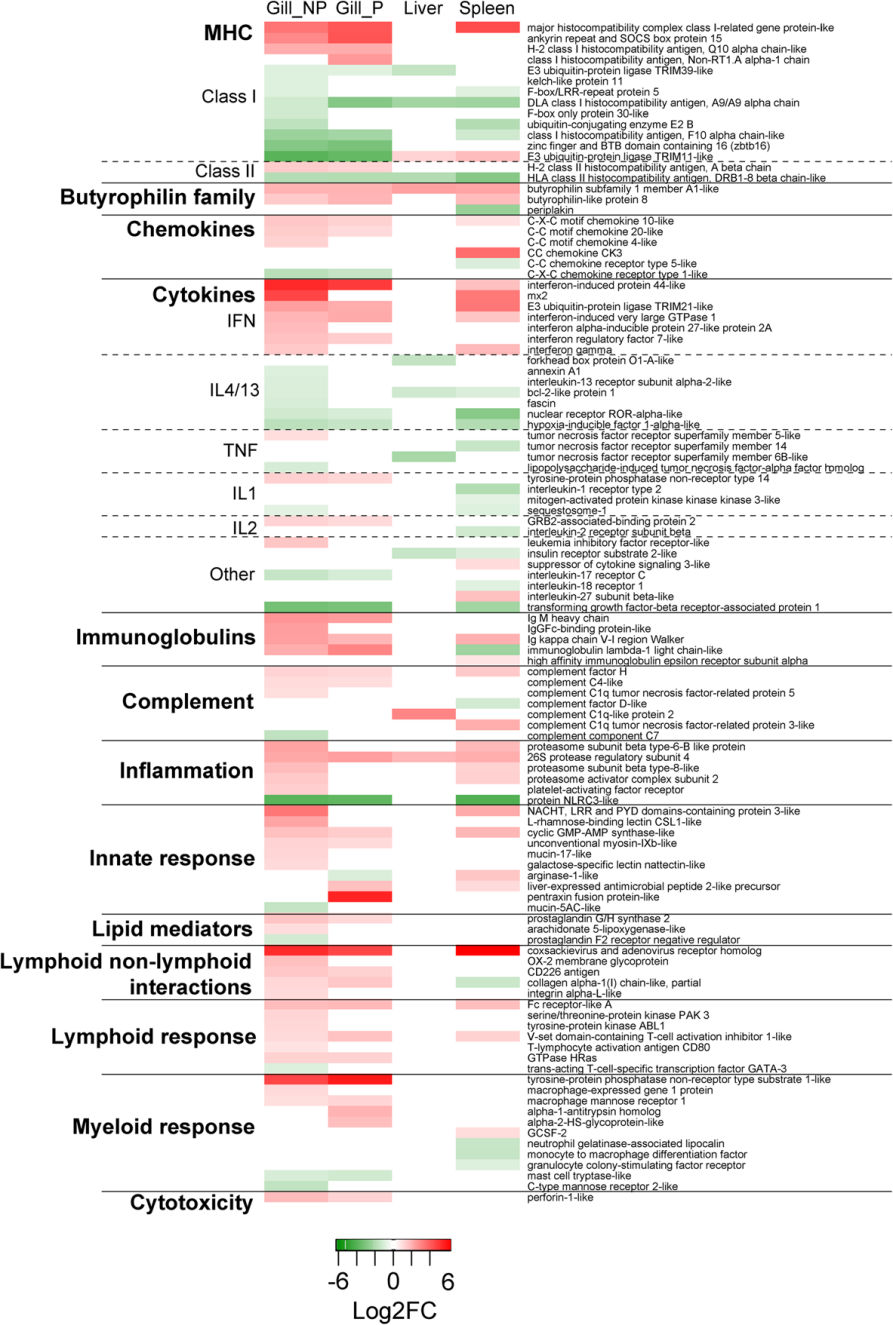
Heatmap depicting the log2 fold change (FC) of the selected genes involved in Immune response. Genes that were not differentially expressed between the infected and the control uninfected groups appear white. Genes that were significantly up- or down-regulated (*q* value < 0.05) by *Sparicotyle chrysophrii* infection appear in shades of red or green (respectively). To facilitate the interpretation of the results genes were grouped in different subcategories. Gill samples were separated in parasitized (P) and non-parasitized (NP) gill sections

To study in detail which genes were regulated by the parasite, we selected DE genes related to the most relevant pathways according to this particular disease: Cell death and proliferation (Fig. 5A), Cellular response to external stimuli (Fig. 5B), Transport of small molecules (Fig. 5C) and Immune system (Fig. 6). Importantly to note, this classification is somewhat arbitrary, as many genes are involved in several pathways or processes, therefore further comments on the subject can be found in the discussion section. Apoptosis markers were clearly up-regulated in the infected gills, particularly in the non-parasitized gills, whereas, in the spleen a more mixed profile was found. All apoptosis inhibitors found were down-regulated, regardless the tissue. Proliferation genes were differentially regulated depending on the tissue and the gene studied (Fig. 5A). Several genes related to response to different stimuli were found to be regulated (Fig. 5B). Interestingly, all hypoxia-related genes were down-regulated in the gills and the spleen of infected GSB, coinciding with the significant down-regulation of *hif1α* in the same tissues inferred by PCR-array. In the liver, a strong down-regulation of genes related to response to stress was observed. P gills exclusively showed an important up-regulation of genes related to response to toxins. Regarding genes involved in transport of ions and iron, a general down-regulation was observed, particularly in NP gills and the spleen (Fig. 5C). Transferrin, however, was significantly up-regulated in P gills. Genes involved in O_2_/CO_2_ transport were also significantly modulated by the infection in NP gills and the spleen.

When studying genes related to the immune response, an important regulation at both, local and systemic levels was observed (Fig. 6). Antigen presentation, particularly MHC class I, was found clearly regulated in the gills, both P and NP. MHC class I antigens were mainly up-regulated, while molecules involved in ubiquitination were down-regulated. Butyrophilin family genes were significantly up-regulated in all studied tissues. All differentially expressed chemokines were up-regulated in the gills and the spleen. Interestingly, chemokine receptors were down-regulated in the same tissues. Interferon and interferon stimulated genes were strongly up-regulated in the gills and the spleen, whereas all differentially expressed genes related to Il4/13 signalling were always down-regulated, regardless of the tissue. A general up-regulation of immunoglobulins, complement, genes related to inflammation, cytotoxicity, innate and adaptive responses was found in infected GSB, particularly in P and NP gills, supporting a strong response of the target tissue upon infection.

## Discussion

The phylum Platyhelminthes (flatworms) is a major subdivision of the animal kingdom, which comprises several groups with parasitic life styles that inflict important diseases in commercial fish [30]. One of them, the Monogenea, is mostly restricted to the skin and gills of marine and freshwater fish. Depending on the configuration of their anchoring apparatus, the monogenean taxonomy splits into Monopisthocotylea and Polyopisthocotylea. The impact on the host depends on the feeding/living strategy, since most monopisthocotyleans feed on mucus and epithelial cells and constantly move around the host surface, whereas most polyopisthocotyleans feed on blood and anchor firmly to the gill lamellae [31, 32]. Fish reaction against monogenean infections has been broached in the last decade using molecular tools. However, most studies have focused on few monogenean genera (all belonging to the Monopisthocotylean clade), mainly *Dactylogyrus* and *Gyrodactylus*, and employed targeted approaches (RT-qPCR) to study few or a group of genes (mainly immune-related genes) [19–27]. To our knowledge, only one microarray study is available in the literature on the host response against a monogenean [33]. Thus far, RNA-seq has been used only recently to analyse the host reaction against some fish parasites [34–38] and no transcriptomic study on the response against a polyopistocothylean has been performed. Therefore, the current study on the reaction of GSB to *S. chrysophrii* is the first comprehensive transcriptomic analysis of a polyopistocothylean infection.

Although the fish used in the current work had a natural mild infection, strong transcriptional changes were detected both at local (gills) and systemic levels (mainly spleen). Interestingly, NP gill portions of infected fish exhibited a higher number of DE genes (1235) than P gill portions (700), indicating a major reaction even in the tissue areas not in contact with the parasite attachment organ, the opistohaptor. The systemic reaction in the spleen was much higher than that at the parasite attachment site, with 1240 DE genes, and by far higher than in the liver (334). This agrees with the fact that the spleen is the largest secondary lymphoid organ of fish [39]. The regulation of genes in lymphohaematopietic organs (spleen and head kidney) has also been reported in other fish-parasites models [40–45], but the local reaction is generally stronger than the systemic. The strong reaction of the spleen in the current study could be due to the hematophagous nature of the parasite, with the consequent changes in erythropoiesis and catabolism of erythrocytes, among others. The liver was included in the current study because of its immunity-associated functions in fish [39, 46], as well as the expected changes in iron metabolism due to the blood-feeding of the parasite. However, the liver responded weakly to the monogenean infection. The only significantly affected pathway was the mitogen activated protein kinases (MAPKs) family signalling cascade.

The fine analysis of the transcriptomic changes by pathways/gene groups allows deciphering the possible mechanisms involved in the host reaction in the different tissues. Apoptosis, cell proliferation and differentiation and autophagy were highly regulated in most organs. All these processes are clearly but complexly interconnected. Many physiological processes, including proper tissue development and homeostasis, require a balance between apoptosis and cell proliferation, since cell-cycle regulators and apoptotic stimuli affect both processes [47]. In the current study, apoptotic markers were up-regulated mostly in NP gills, and down-regulated mainly in the spleen, whereas markers of inhibition of apoptosis were down-tuned in most tissues. The splenic inhibition of apoptosis was mainly due to the suppression of BH3-only proteins and tumour suppressor TP53 (*p53*). While the latter exerts its tumour suppressive role in part by regulating transcription of a number of genes involved in apoptotic cell death [48], BH3-only proteins are considered cytosolic sentinels selectively triggering apoptosis in response to developmental cues or stress-signals like DNA damage [49]. Apoptosis is a cascade of genetically controlled events that lead to cell death, induced by many different signals [50]. Parasite-induced apoptosis is a complex interaction specific to each parasite-host system, influenced by whether the parasite has evolved mechanisms that induce or avoid host cell apoptosis to enable its survival [51]. The timing of apoptosis seems to be crucial to determine its outcome, since in early onset it contributes to destruction of intracellular parasites, and favours the penetration of multicellular parasites. In contrast, the late apoptosis of engaged immune cells, suppresses the detrimental effect of the over-inflammatory reaction benefiting the host, but also the parasite as it sets the milieu for its survival [52]. In the *Sparicotyle* infection model, late apoptosis is the most probable scenario in gills, as immune effectors are already engaged, suggesting a two-edged sword effect: counteraction of the inflammatory reaction that helps to preserve tissue integrity, but serves the monogenean survival as well. Further experimental infections with controlled timings and comparison of data from severe parasite loads will help to elucidate this.

Markers of cell proliferation/differentiation had varied patterns of changes, though it seems that these pathways were globally enhanced in both types of gill tissues, as previously seen in the PCR-array, with increased levels of *pcna* (in P gills) and *tgfβ* (in P and NP gills). This could be explained by a need to repair the damage induced by the parasite in the gill epithelium. On the other hand, this enhanced proliferation could also be an apoptotic stimulus, as uncontrolled proliferation can be associated with a high level of apoptosis [47]. In the spleen, there was an up-regulation of genes involved in pathways that support proliferation and activation of cell lineages. Among them, the most highly up-regulated pathway was that of polyamines metabolism. In mammals, polyamines, ubiquitous small basic molecules such as putrescin, spermidine and spermine, are involved in modulation of chromatin structure, gene transcription and translation, DNA stabilization, signal transduction, cell growth and proliferation, migration, membrane stability, functioning of ion channels and receptor-ligand interactions [53]. Polyamines are tightly regulated because a decrease in their concentrations inhibits cell proliferation, while an excess appears to be toxic. In fish, spermidine has been shown to protect against oxidative stress in inflammation models using macrophages [54]. Interestingly, also in GSB, polyamine markers were up-regulated in the intestine of fish challenged with the parasitic myxozoan *Enteromyxum leei* [55]. Thus, the observed major up-regulation of genes involved in polyamines metabolism in the spleen could be a systemic response aiming to protect against the inflammatory response generated by the *S. chrysophrii* infection.

Autophagy, with a clear down-regulation pattern in the spleen in the current study, is an essential homeostatic process of cell components breakdown, enabled by the lysosomal degradation pathway, mostly under circumstances of nutrient deprivation, or as aftermath of dangerous stimuli such as infection [56]. However, the intricate interaction between autophagy and immunity and inflammation is complex, since autophagy proteins function in both the induction and suppression of immune and inflammatory responses, and immune and inflammatory signals function in both the induction and suppression of autophagy [57]. Autophagy proteins have been involved in some parasite models [58], suggesting that they aid the host in the removal of invading pathogens [56]. In humans, the inhibition of macroautophagy has been observed in response to cytokines associated primarily with humoral immune response, particularly IL4 and IL13 [59]. Since the pathway components of the latter were also down-regulated in our study, these cytokines could not be accounted for the down-regulation of the macroautophagy, therefore this observation warrants further research.

The MAPKs pathway had a differential regulation depending on the tissue: it was down-regulated in the liver, but up-regulated in NP gills and the spleen. The up-regulation was in part due to some proteasome components. The proteasome complex plays a key role in the maintenance of protein homeostasis by removing misfolded or damaged proteins, which could impair cellular functions, and by removing proteins whose functions are no longer required. Therefore, the proteasome participates in numerous cellular processes, including cell cycle progression, apoptosis, or DNA damage repair [60]. MAPKs are conserved serine/threonine kinases involved in the response to many extracellular stimuli like mitogens, osmotic stress, heat shock and proinflammatory cytokines. They transduce the signal from the cell surface to the DNA, orchestrating intracellular processes such as gene expression, metabolism, proliferation, differentiation, mitosis, cell survival and apoptosis [61]. Thus, they are key regulators of cellular physiology and immune responses, and abnormalities in MAPKs are implicated in many diseases in humans and mammals [61–63]. Some human parasites manipulate host signalling pathways and the innate immune system to establish infection, specifically targeting different mechanisms within the MAPK cascade [64]. In the current fish-parasite model, the increase of the MPAKs pathway supports the need to maintain homeostasis in all the tissues to face the parasite damage and the apoptotic/inflammation/proliferation-triggered pathways. In addition, the enhanced expression of the *26S protease regulatory subunit 4* (a component of the 26S proteasome, a multiprotein complex involved in the ATP-dependent degradation of ubiquitinated proteins) in all tissues, prompts us to consider the ubiquitin–proteasome mechanism, the main enhanced way of cellular protein degradation, since the alternative way, the autophagy–lysosome system, was down-regulated. Whether this regulation is due to a direct regulatory mechanism of the parasite or it is simply induced by all the apoptosis and regeneration processes occurring during the pathology remains to be determined.

Other pathways affected by the infection were related to oxidative stress, stress, hypoxia, O_2_/CO_2_ transport, ionic balance and iron metabolism. Interestingly, most of them were not only down-regulated locally, as expected due to the nature of parasite infection, but also in the spleen, which seems to be translating the local effects of haemolysis, anaemia, reduced oxygen availability, epithelial cell damage and inflammation. All this down-regulation could be interpreted as a shutdown of the metabolism, like a hypometabolic response, to counteract the low oxygen levels induced by the parasite damage to the gills and to concentrate the energy on the immune response. Among the hypoxia-related genes, special attention must be paid to HIF, which mediates the adaptation of cells and tissues to low oxygen concentrations. It is a heterodimeric transcription factor that is composed of a hypoxia-inducible alpha subunit (HIF1α and HIF2α) and a constitutively expressed beta subunit (HIF1β). We detected a strong down-regulation of *hif1α* in all the tissues both in the RNA-seq analysis and in the preliminary PCR-array (Additional file 1). Many studies revealed that hypoxia in fish often results in an elevated transcription of *hif* genes. However, a convincing explanation for the activation of *hif* transcription in teleost fish under hypoxic conditions has not yet been presented [65]. Different fish species exposed to experimental acute or chronic hypoxia, displayed significant changes in the expression of oxidative stress genes not only in the gills, but also in other organs including the spleen, generally having *hif1* up-regulated [66, 67]. However, depending on the duration of the hypoxia, some *hif* subunits can be significantly down-regulated in some lymphoid tissues, as the head kidney [68]. In the current fish-parasite model, although we do not know the initial time of the infection, we could hypothesize that the hypoxia induced by the parasite is consequence of a long-term infection, and therefore the observed down-regulation of *hif1α* is probably part of the mentioned hypometabolism, as the ultimate defence in stress response [69]. In fact, we recently demonstrated that GSB is a highly euryoxic fish, capable of adjusting the mitochondrial machinery to cope with a decreased basal metabolic rate, when hypoxia-challenged [70]. The concomitant down-regulation of the *hif1α* inhibitor in the current study could help to tolerate the hypoxia, as this was previously demonstrated experimentally in zebrafish, when the *fih* gene encoding an inhibitor of hypoxia-inducible factors was deleted [71].

The only iron-related up-regulated gene was transferrin in P gills. Other iron-related genes, such as ferritin, were down-regulated in NP gills and the spleen (Fig. 4C). Transferrin is an iron-binding glycoprotein involved in the transport and storage of iron, it participates in the regulation of breathing, cell proliferation and differentiation, and also plays an important role in host defence against pathogenic infection, not only because it inhibits the growth and proliferation of pathogens, but it also activates anti-microbial responses in macrophages [72]. Similarly, the expression of transferrin was significantly up-regulated in other fish species exposed to bacterial [73] and parasitic [74] pathogens, in the latter case also in gills. The higher expression of transferrin in P gills could correlate with a higher need of iron to transport the low oxygen available or alternatively it could be an effort to increase iron storage to deprive its availability for the parasite.

When analysing immune-related pathways, both P and NP gills were the most enriched tissues, followed by the spleen and the liver. The general overview could be considered as an inflammatory scenario in the gills, with a clear up-regulation of some pro-inflammatory cytokines, such as *ifn*γ, IFN-regulated genes and other inflammation markers, and activation of immunoglobulins, complement factors, lymphoid and myeloid response, some lectins and cytotoxic enzymes as perforin-1-like. In most monogenean studies focusing on the regulation of immune genes, *il1β*, *tnfα* [19, 20, 23, 25–27, 75], *ifnγ* [25, 76], *tgfβ* [19, 20, 26, 27, 75] and some TLRs [22] appeared up-regulated at the local level (gills, skin) in relatively short times after exposure to the parasite (from 4 to 30 days). We also observed a down-regulation of several molecules related to the Il4/13 signalling pathway and down-regulation of mucin 5 in NP gills, which is opposite to what we recently described in Atlantic salmon (*Salmo salar*) infected with the gill parasite *Neoparamoeba perurans* [77]. Thus, the present results are another proof of the relationship between mucin 5 and type 2 cytokines, and the importance of their regulation during respiratory disorders, as it has also been described for mammals [78]. The up-regulation in spleen of regulatory proteins such as *il27β* (*ebi3*), *socs3* or *il10* (the latter only detected in the preliminary PCR-array) could indicate an attempt to balance protective and pathological effect of immunity. However, to reach stronger conclusions further studies in more controlled scenarios have to be conducted.

In our preliminary PCR-array, we detected *c3* down-regulation in the spleen, which would be congruent with the low complement levels detected in the serum of *Sparicotyle*-infected GSB [16]. By contrast, other complement factors detected in the RNA-seq study were up-regulated locally. The complement system exerts a significant evolutionary pressure on pathogens. In consequence, parasites have evolved several mechanisms to surpass or inhibit the complement system, which is of particular importance in hematophagous parasites [79]. One hypothesis that arises from the current results is the possible use of complement factor H (FH), up-regulated in the gills and the spleen, as an evasion strategy. FH is a negative regulator of complement used to protect host tissues from aberrant complement activation. In the few monogenean studies in which *c3* was included, it appeared also down-regulated locally [20, 23] and some human parasites use FH to down-regulate the alternative pathway complement [80].

The most down-regulated gene in the gills and the spleen was *nlrc3-like*. NOD-like receptors (NLRs) are a large group of cytoplasmic receptors that play an important role in detecting and responding to a large range of pathogen- and damage-associated molecular patterns (PAMPs and DAMPs) in higher vertebrates. They are part of the inflammasome, large intracellular multiprotein complexes that play a central role in innate immunity. NLRC3 in particular, is a cytoplasmic protein that negatively regulates pro-IL1β maturation and can interact with apoptosis. Overexpressed NLRC3 acts as an anti-inflammatory cytosolic protein in mammals [81]. Although the immunological significance of Nlrc3 protein in fish remains largely uncharacterized, it seems it could have similar functions, as it is ubiquitously expressed in many fish tissues [82–84]. Furthermore, in rainbow trout the up-regulation of *nlrc3* expression in response to bacterial LPS injection was considered to attenuate the PAMPs-induced inflammatory response [84]. Thus, the *nlrc3* down-regulation in the current study could also be interpreted as an inflammation force.

Other immune genes interesting to denote are *coxsackievirus and adenovirus receptor homolog* (*cxadr*) and members of the butyrophilin family, which were up-regulated both in the gills and the spleen. CXADR (= CAR) is a component of the epithelial apical junction complex. It is essential for tight junctions integrity and is also involved in transepithelial migration of leukocytes. It is predominantly expressed in endothelial and epithelial cells, helps to establish cell polarity and provides a barrier function, regulating migration of immune cells in mammals [85]. Interestingly, *cxadr* was also up-regulated in the intestine of GSB fed with butyrate-supplemented diets [86], known to strengthen the epithelial barrier and reduce inflammation [87]. Therefore, the observed increase of transcripts in infected fish could be reflecting the need to strengthen the gill epithelium to face the damage induced by the parasite and to favour the transport of leukocytes from the spleen. Butyrophilins (BTNs) and butyrophilin-like (BTNL) molecules are regulators of immune responses that belong to the immunoglobulin superfamily. BTNs are implicated in T cell development, activation and inhibition, as well as in the modulation of the interactions of T cells with antigen presenting cells (APCs) and epithelial cells. In particular, *Btnl1* mRNA is broadly expressed in mouse APCs, and has been recognised as an inhibitor of T cell activation [88]. In fish, information on the function of this family is very scarce, but it seems to be in line with that in mammals, as LPS challenge up-regulated the expression of *btn1a1* in the intestine of Asian sea bass (*Lates calcarifer*) [89]. In our study, *btnl1* was significantly up-regulated even in the liver. This increased expression of *btnl1* would be in agreement with the increased levels of some MHC class II markers in the gill, as exogenous antigens are phagocytosed or endocytosed by APCs, degraded, and presented on MHC class II molecules to CD4^+^ T cells [90]. In addition, few MHC class I markers, which are related with the degradation of self-antigens, were also increased in the gills. This agrees with the enhanced inflammation, cellular proliferation and apoptosis observed in the gills, but not in the spleen.

## Conclusions

By using comparative transcriptomic analysis, we have identified a total of 2581 DE genes in GSB naturally infected with a flatworm. Functional annotation of these genes clearly showed a different pattern of gene expression of the different GO categories depending on the fish tissue, with contrasting forces acting at the local and systemic level. Apoptosis, inflammation and cell proliferation dominate the gills, whereas inhibition of apoptosis, autophagy, platelet activation, signalling and aggregation, circadian clocks and inflammasome were observed in the spleen. Proteasome markers were increased in all tissues, as an effort to maintain homeostasis to face the parasite damage and the host triggered pathways. All this down-regulation could be part of a hypometabolic response, to counteract the hypoxia induced by the parasite damage to the gills and to concentrate the energy on the defence and repair mechanisms. Alternatively, this splenic down-regulation could be interpreted as the often observed action of helminths striving to modify host immunity in the interest of their own longevity, reproduction and persistence [91].

## Methods

### Animals and samplings

A total of 20 gilthead sea bream (GSB) juveniles (weight = 90 ± 29.22 g, fork length = 17.3 ± 1.73 cm) were used in this study. The fish were obtained from a stock with a mild *Sparicotyle chrysophrii* natural infection (intensity = 2.73 parasites/fish; prevalence = 55% from *n* = 100 sampled fish), sampled in mid-November 2016 (water temperature = 17–19 °C) from a sea cage facility at East Adriatic Sea coast (Croatia). The fish were introduced in the sea cages on March of the same year. Thus, although the exact timing of infection cannot be known, we can assume this is a long-term infection probably initiated some months after introduction, with the rise of sea water temperature. Fish were removed from the cages, and gill arches were firstly visually inspected. Ten animals with gross changes in gills and presence of monogeneans were placed in a bath with anaesthetic overdose (MS-222, 0.1 g/l), euthanized and immediately dissected under the stereomicroscope (infected group). In parallel, 10 fish without infection were euthanized, gills dissected and controlled under the stereomicroscope for the absence of any monogenean developmental stages (control group). From each infected (INF) fish, a piece of gill tissues in direct contact with the monogenean (P, parasitized) was dissected after detaching the parasite, and another piece of non-parasitized (NP) gill tissues from the same gill arch, but opposite gill chamber, were placed in RNAlater. Control gill tissues were obtained from uninfected fish (CTRL). Spleen and liver samples were also collected in RNAlater from INF and CTRL fish. Fish were also checked for potential coinfections with other parasites by standard diagnostic protocols and tested negative.

### RNA extraction

Pieces of spleen, liver and gill filaments (without cartilage) were removed from the RNAlater and approximately 100 mg of tissue were homogenized in 1 ml of TRI Reagent solution from the MagMAX™-96 for Microarrays Total RNA Isolation Kit (Applied Biosystems, Foster City, CA, USA) that was used for subsequent RNA isolation following the manufacturer’s instructions. The RNA concentration and purity was determined using a Nanodrop 2000c (Thermo Scientific, Wilmington, DE, USA). Quality and integrity of the isolated RNA were checked on an Agilent Bioanalyzer 2100 total RNA Nano series II chip (Agilent, Amstelveen, Netherlands). RNA integrity number (RIN) values were between 8 and 10.

### Reverse transcription and gene expression analyses

A preliminary targeted PCR-array was performed on gill and spleen tissues of the 10 uninfected control fish (CTRL) and the 10 *Sparicotyle* infected fish (INF) to assess the magnitude of the response and determine the best responders and best quality samples to be selected for Illumina RNA sequencing. Reverse transcription of 500 ng of input RNA was performed using the High-Capacity cDNA Archive Kit (Applied Biosystems, Foster City, CA, USA) following the manufacturer’s instructions. Negative control reactions were performed excluding the reverse transcriptase. A 96-well PCR-array layout was used for the simultaneous profiling under uniform conditions of 45 selected genes. The selected genes and primers used are shown in Additional file 1B and include genes involved in response to hypoxia, apoptosis, cell growth and regeneration, inflammation, iron metabolism, tight junction proteins, cytokines, immunoglobulins, antioxidant markers, mucins, antiproteases, complement, acute phase proteins, pathogen recognition receptors, lymphocyte markers and MHC proteins. The primers were designed and checked for specificity using the GSB transcriptomic database [92] (http://nutrigroup-iats.org/seabreamdb/). All primers had similar efficiencies higher than 90% and produced amplicons of 50–150 bp. Each 25 μl PCR reaction contained 660 pg of total input RNA, 12.5 μl of 2× SYBR Green Master Mix (BioRad, Hercules, CA, USA) and 0.9 μM of specific primers. Liquid manipulations needed to construct the PCR array were performed using an EpMotion 5070 Liquid Handling Robot (Eppendorf, Hamburg, Germany) which allowed for the precision needed to avoid the use of technical replicates. The real-time quantitative PCR (RT-qPCR) was carried out using an Eppendorf Mastercycler EP Realplex Real-Time PCR detection system (Eppendorf). The PCR amplification program consisted of an initial step at 95 °C for 3 min, followed by 40 cycles of denaturation at 95 °C for 15 s and annealing/extension at 60 °C for 60 s. The efficiency of all runs was checked to be always higher than 90% and the specificity was verified by analysis of melting curves. Fluorescence data acquired during the extension phase were normalized by the delta-delta Ct method [93] using *β-actin* as a housekeeping gene. *β-actin* was selected as the housekeeping gene because it showed to be the more stable reference gene among conditions in each tissue using the GeNorm software for stability testing. The other reference genes tested were *elongation factor 1α*, *α-tubulin* and *18S rRNA*. Statistical differences (*p* < 0.1 and *p* < 0.05) between CTRL and INF samples for each primer and tissue were determined by Student’s *t* test when samples were normally distributed or non-parametric Mann-Whitney-Wilcoxon test when normality conditions were not met.

### Illumina sequencing

Using the preliminary PCR-array results, four CTRL and five INF fish were selected for Illumina sequencing. Spleen, liver and gill samples were included from each selected fish. In infected fish, the gills were divided in two independent samples: gill portions were the parasite was present (parasitized, P) and gill portions without parasite presence (non-parasitized, NP). This yielded a total of 32 samples. Illumina RNA-seq libraries were prepared from 500 ng total RNA using the Illumina TruSeq™ Stranded mRNA LT Sample Prep Kit (Illumina Inc. San Diego, CA, USA) according to the manufacturer’s instructions. All RNA-seq libraries (150–750 bp inserts) were sequenced on an Illumina HiSeq2500 sequencer as 1 × 50 nucleotides single-end reads according to the manufacturer’s protocol. Image analysis and base calling were done using the Illumina pipeline.

### Bioinformatic analyses and sample quality assessment

Approximately ~ 650 million single-end reads were obtained from the 32 samples sequenced with an average of 20.5 million reads per sample. Quality analysis was performed with FastQC v0.11.7 (https://www.bioinformatics.babraham.ac.uk/projects/fastqc/) and libraries were filtered with Prinseq [94] for quality > 30 and < 10% of Ns in the sequence. Afterwards the libraries were mapped and annotated using Tophat2 [95] and the *Sparus aurata* draft genome as a reference (http://www.nutrigroup-iats.org/seabreamdb/). Non-annotated transcripts were subjected to a second annotation round using the NCBI tool blastx [96] with an *e*value of 10e^− 5^ as cutoff threshold and the NCBI non-redundant (NR) database. A representative transcriptome per sample was constructed using Cufflinks [97]. Before proceeding with the differential expression analyses Cufflinks data were quality checked using cummeRbund [97]. Three samples that yielded anomalous FPKM density distributions (one spleen, one P gill and one NP gill, all from INF fish) were removed (Additional file 4).

### Differential expression analyses, statistics and visualizations

Differentially expressed transcripts were obtained using Cuffdiff [97]. Comparisons were performed for each infected tissue (liver *n* = 5, gills/spleen *n* = 4) against their corresponding uninfected control group (*n* = 4 in all tissues). Differentially expressed (DE) transcripts were considered at *q* value < 0.05. Log2 fold change values were used to separate up- and down-regulated transcripts. The lists of annotated total, up- and down-regulated genes in each tissue were used to perform gene ontology (GO) analysis using the R package GOseq [98, 99] taking into account the gene size in our particular species to avoid biases, and using the Wallenius approximation. Significantly enriched GO categories were obtained after FDR correction using a cutoff of 0.05 [100]. All tools mentioned above are contained in the GPRO suite [101]. Pathway analyses were performed using the Reactome online tool and the ReactomePA R package [102, 103]. Of note, Reactome, just like other available pathway analysis tools, is a database mainly based on human genes and pathways and does not contain GSB information. This analysis was therefore performed after converting the GSB identifiers into their human equivalents, when possible. Thus, we are aware that this pathway analysis is not complete, but can still yield interesting hints to navigate this massive amount of data in a more comprehensive way. Pathway enrichment analysis used the hypergeometric model [104] to calculate *q* values. Hierarchical clustering and principal component analysis using the FPKM values for all DE transcripts were performed using the factoextra R package [105] and the default setting of the functions unless otherwise stated. Heatmaps were constructed using the heatmap.2 R function and the log2 fold change of the selected genes.

## Additional files

Additional file 1: A) PCR-array results: Mean and SEM of the fold changes of the 45 selected genes in *Sparicotyle* infected fish (*n* = 10) calculated relative to the control uninfected group (*n* = 10) in parasitized gill portions (with parasite presence), non-parasitized gill portions (no parasite present) and spleen. Student’s *t* test or Mann-Whitney-Wilcoxon test when normality conditions were not met were used to calculate *p* values comparing expression levels of control and infected animals per gene. Dark red and green indicate significantly up- or down-regulated genes, respectively, with *p* < 0.05. Light red and green indicate up- or down-regulated genes, respectively, with *p* < 0.1. The first column (Pathway) indicates the main pathway with which each gene is related. These pathways were targeted due to the nature of the specific disease studied. B) Information on the primers used in this study. (PDF 708 kb)

Additional file 2: Gene ontology (GO) enrichment analysis based on the number of DE genes per tissue (FDR cutoff 0.05). The Tabs entitled “TISSUE EnrichGO” contain the detailed lists of the enriched GO total, up- and down-regulated sorted by ontology (Biological process (BP), Cellular compartment (CC) and Molecular function (MF)). (XLSX 49 kb)

Additional file 3: Pathway classification of DE genes. Bar plots representing the percentage of proteins involved in each broad Reactome pathway. The column labelled Reactome was added as a reference to show the percentage of genes in each category in the database. The other columns represent the percentage of differentially expressed (DE) genes in each category when considering all DE genes in all the tissues of the current experiment (Total) or per tissue, when compared to the control uninfected group. Gill samples are separated in parasitized (P) and non-parasitized (NP) gill sections. (PDF 343 kb)

Additional file 4: Exploratory analysis of the samples prior to differential expression study. The FPKM density distributions showed three samples with anomalies (red arrows and red in the legend) that were not included in downstream analyses: gill infected parasitized 1 (A), gill infected non-parasitized 5 (B) and spleen infected 2 (D). All liver samples (C) showed uniform distributions and were included in the analyses. (PDF 292 kb)

## Abbreviations

CTRL: Control fish with no *S. chrysophrii* infection
DE: Differentially expressed
FDR: False discovery rate
GO: Gene ontology
GSB: Gilthead sea bream
Hb: Haemoglobin
INF: Fish infected with *S. chrysophrii*
NP: Non-parasitized, gill portions from infected fish with no parasite attached
P: Parasitized, gill portions where the parasite was attached
PCA: Principal component analysis
