# Supplementary material for: Acting locally - affecting globally: RNA sequencing of gilthead sea bream with a mild Sparicotyle chrysophrii infection reveals effects on apoptosis, immune and hypoxia related genes
Source: BMC Genomics. 2019 Mar 11;20:200. doi: 10.1186/s12864-019-5581-9 (PMC6416957; doi:10.1186/s12864-019-5581-9)
Supplement: Supplementary file 1 — A) PCR-array results: Mean and SEM of the fold changes of the 45 selected genes in Sparicotyle infected fish (n = 10) calculated relative to the control uninfected group (n = 10) in parasitized gill portions (with parasite presence), non-parasitized gill portions (no parasite present) and spleen. Student’s t test or Mann-Whitney-Wilcoxon test when normality conditions were not met were used to calculate p values comparing expression levels of control and infected animals per gene. Dark red and green indicate significantly up- or down-regulated genes, respectively, with p < 0.05. Light red and green indicate up- or down-regulated genes, respectively, with p < 0.1. The first column (Pathway) indicates the main pathway with which each gene is related. These pathways were targeted due to the nature of the specific disease studied. B) Information on the primers used in this study. (PDF 708 kb) [file 12864_2019_5581_MOESM1_ESM.pdf]

Additional file 1:

A) PCR-array results: Mean and SEM of the fold changes of the 45 selected genes in *Sparicotyle* infected fish (n = 10) calculated relative to the control uninfected group (n = 10) in parasitized gill portions (with parasite presence), non-parasitized gill portions (no parasite present) and spleen. Student's t test or Mann-Whitney-Wilcoxon test when normality conditions were not met were used to calculate p values comparing expression levels of control and infected animals per gene. Dark red and green indicate significantly up- or down-regulated genes, respectively, with $p < 0.05$. Light red and green indicate up- or down-regulated genes, respectively, with $p < 0.1$. The first column (Pathway) indicates the main pathway with which each gene is related. These pathways were targeted due to the nature of the specific disease studied.

		Parasitized gills			Non-parasitized gills			Spleen		
Pathway	Gene	Fold change	SEM	*p*	Fold change	SEM	*p*	Fold change	SEM	*p*
Hypoxia	*hif1α*	0.675	0.098	0.045	0.693	0.054	0.016	0.700	0.075	0.049
Inflammation	*anxa1*	0.840	0.044	0.160	0.644	0.070	0.016	-	-	-
Iron metabolism	*hepc*	1.618	0.425	0.170	4.040	2.996	0.350	1.596	0.455	0.240
	frim	0.916	0.139	0.670	0.628	0.118	0.020	0.704	0.104	0.160
Tight junction proteins	*ocln*	1.500	0.270	0.080	0.973	0.186	0.740	-	-	-
	cldn12	0.845	0.123	0.250	0.750	0.066	0.015	-	-	-
	cldn15	1.151	0.147	0.300	1.537	0.164	0.010	-	-	-
	tjp1	0.932	0.153	0.650	0.796	0.056	0.030	-	-	-
Cytokines	*il1β*	0.603	0.114	0.360	0.899	0.231	0.600	1.467	0.220	0.028
	il6	0.636	0.127	0.110	0.732	0.118	0.200	1.137	0.114	0.430
	il8	0.982	0.133	0.280	1.303	0.349	0.480	1.986	0.377	0.033
	il10	1.083	0.134	0.600	1.034	0.159	0.730	1.384	0.088	0.014
	tnfα	1.028	0.166	0.430	1.233	0.198	0.190	2.260	0.730	0.009
Immunoglobulins	*sIgM*	1.390	0.258	0.160	1.472	0.191	0.060	1.234	0.207	0.370
	sIgT	0.375	0.365	0.120	0.490	0.340	0.390	0.793	0.072	0.140
	mIgT	1.141	0.253	0.620	1.295	0.210	0.320	1.180	0.160	0.430
	mIgM	0.870	0.128	0.460	1.289	0.307	0.400	1.273	0.191	0.240
	IgD	1.154	0.213	0.510	1.287	0.334	0.450	-	-	-
Complement	*c3*	-	-	-	-	-	-	0.670	0.101	0.040
Lymphocyte markers	*CD4-1*	0.955	0.168	0.780	0.815	0.064	0.060	1.110	0.132	0.540
	CD8α	1.321	0.174	0.080	1.340	0.142	0.060	1.507	0.137	0.007
	CD8β	1.401	0.193	0.040	1.353	0.196	0.130	1.374	0.127	0.041
	zap70	1.000	0.045	0.830	0.986	0.081	0.890	1.127	0.093	0.180
	prf1	1.036	0.078	0.420	1.100	0.156	0.560	1.218	0.249	0.650
MHC markers	*mhcIIb*	1.122	0.201	0.610	1.073	0.117	0.370	1.208	0.073	0.020
	β2m	0.828	0.048	0.052	0.820	0.081	0.054	0.944	0.133	0.110
Antiproteases	*a2m*	1.105	0.283	0.730	0.763	0.127	0.270	0.497	0.093	0.007
Acute phase proteins	*ctsb*	0.855	0.047	0.140	0.924	0.058	0.390	1.009	0.064	0.920
	ctsl	0.827	0.078	0.210	0.784	0.117	0.020	0.953	0.092	0.760
Pattern recognition receptors	*tlr2*	0.984	0.160	0.920	1.133	0.169	0.510	1.021	0.093	0.840
	tlr9	0.823	0.142	0.270	0.804	0.058	0.060	1.080	0.143	0.650
	clec10a	1.278	0.362	0.380	1.053	0.121	0.740	1.279	0.140	0.101
	lgals1	0.870	0.128	0.620	0.965	0.100	0.560	1.958	0.414	0.034
	lgals8	0.802	0.042	0.018	0.770	0.063	0.036	0.911	0.083	0.400
	fcl	0.766	0.386	0.350	0.994	0.373	0.320	1.351	0.844	0.790
Mucins	*imuc*	1.242	0.093	0.220	2.136	0.768	0.120	-	-	-
	muc18	1.099	0.077	0.360	0.951	0.049	0.550	-	-	-
Apoptosis	*casp3*	1.020	0.257	0.140	0.729	0.039	0.002	0.806	0.029	0.018
Antioxidant markers	*cat*	0.830	0.176	0.270	0.847	0.070	0.170	0.793	0.064	0.320
	sod2	0.759	0.125	0.090	0.785	0.067	0.040	0.991	0.138	0.950
	gst3	1.179	0.220	0.430	0.941	0.141	0.540	1.008	0.079	0.950
	hsp70	0.703	0.163	0.080	0.712	0.042	0.004	0.931	0.082	0.600
	hsp90α	1.562	0.156	0.045	0.997	0.235	0.960	0.725	0.193	0.960
Cell growth & regeneration	*pcna*	1.465	0.170	0.003	1.303	0.138	0.004	-	-	-
	tgfβ	1.265	0.113	0.056	1.662	0.140	0.001	1.071	0.086	0.540

Additional file 1:

B) Information on the primers used in this study.

Pathway	Gene name	Symbol	GenBank	Forward primer (5'-3')	Reverse primer (5'-3')
Hypoxia	Hypoxia inducible factor-1 alpha	*hif1α*	JQ308830	CAGATGAGCCTCTAACTTGTGGAC	TTAGCAAGAATGGTGGCAAGATGAG
Inflammation	Annexin A1	*anxa1*	MF979884	GATGTGGTGCTGGCTCTG	TGTGCCGAGTCCCTTCATAG
Iron metabolism	Hepcidin	*hepc*	AM749960	ACTCCTGGAAGATGCCGTATGC	AACTTACACCTCCTGCGTCCAC
	Ferritin	*frim*	MF979883	GCCGAGAAGCTGCTGTCCTT	CACTCATCACGCTCTGGTTTCTTGAC
Tight junction proteins	Occludin	*ocln*	KF861990	GTGTCAGAACCTCTACCAGACCAGCTACTC	GAAAGCCTCCCACTCCTCCCATCT
	Claudin-12	*cldn12*	KF861992	CTCTCAGGGCTACACATCTACCTATGC	ACATTCGTGAGCGGCTGGAG
	Claudin-15	*cldn15*	KF861993	CCGATTGTGGAAGTAGTGGCTCTGGT	CAGCATCACCCAACCGACGAACC
	Tight junction protein ZO-1	*tjp1*	KF861994	AAGCAGTATTACGGTGACTCA	TGCATCCCTGGCTTGTAG
Cytokines	Interleukin-1 beta	*il1β*	AJ419178	GCGACCTACCTGCCACCTACACC	TCGTCCACCGCCTCCAGATGC
	Interleukin-6	*il6*	EU244588	TCTTGAAGGTGGTGCTGGAAGTG	AAGGACAATCTGCTGGAAGTGAGG
	Interleukin-8	*il8*	JX976619	CAGCAGAGTCTTCATCGTCACTATTG	AGGCTCGCTTCACTGATGG
	Interleukin-10	*il10*	JX976621	AACATCCTGGGCTTCTATCTG	GTGTCCTCCGTCTCATCTG
	Tumor necrosis factor-alpha	*tnfα*	AJ413189	CAGGCGTCGTTCAGAGTCTC	CTGTGGCTGAGAGCTGTGAG
Immunoglobulins	Immunoglobulin M soluble form	*sIgM*	JQ811851	ACCTCAGCGTCCTTCAGTGTTTATGATGCC	CAGCGTCGTCGTCAACAAGCCAAGC
	Immunoglobulin T soluble form	*sIgT*	KX599200	GCTGTCAAGGTGGCCCCAAAAG	CAACATTCATGCGAGTTACCCTTGGC
	Immunoglobulin T membrane-bound form	*mIgT*	KX599201	AGACGATGCCAGTGAAGAGGATGAGT	CGAAGGAGGAGGCTGTGGACCA
	Immunoglobulin M membrane-bound form	*mIgM*	KX599199	GCTATGGAGGCGGAGGAAGATAACA	GCAGAGTGATGAGGAAGAGAAGGATGAA
	Immunoglobulin D	*IgD*	MF974245	GACCCACAGCAAACCTTCAG	AGAGACCAGGCACACGATT
Complement	Complement factor C3	*c3*	HM543456	GCTTACGCTCTTCTTGCTCTGGTGAA	CATCTGACAACTGGTCTGGCATCGT
Lymphocyte markers	CD4-1	*CD4-1*	AM489485	TCCTCCTCCTCGTCCTCGTT	GGTGTCTCATCTTCCGCTGTCT
	Cluster of differentiation 8 alpha	*CD8α*	EU921630	GCAGCAACGGTAACACGAACG	CCAGTATGAGCGGAGTACAGAACA
	Cluster of differentiation 8 beta	*CD8β*	KX231275	CCGAAATGTGGAAGACTGGAACTC	CTTTGGAGGTAAGGTTGGAGGGAT
	Zeta-chain-associated protein kinase 70	*zap70*	MF175239	TGGTGAAGGAGGAGATGATGAGG	GCGAACGATGTAGCGGTTGT
	Perforin	*prf1*	MF175233	ACGGATGGCTATGTGAAGGTGTC	TGTGGGTTGTTGTTGTTGTGAATGA
MHC markers	MCH class II beta	*mhcIIb*	MF979882	CCTCTGGTGACGGACTGG	CGATGGCGATCTTGTTTCTCTCA
	Beta 2 microglobulin	*β2m*	MF979881	GGCACTTCCATCTGACCAAGA	GCTGAACCGCTCTCCACG
Antiproteases	Alpha-2-macroglobulin	*a2m*	AY358020	TCCTGGGTGACATTCTGGGT	CCGTATGGCATCCTCAGCAG
Acute phase proteins	Cathepsin B	*ctsb*	KJ524457	TGATTCCCATGTCGGTTGTC	GGGTCTACTGCCATTCACAT
	Cathepsin L	*ctsl*	KM522787	GGGAACGGATGACCAGCCTTGT	CGGTGTCATTGGCAGAGTTGTAGTTG
Pattern recognition receptors	Toll-like receptor 2	*tlr2*	KF857323	CATCTGCGACTCTCCTCTCTTCCT	ATTCAACAATGGAGCGGTGGACTT
	Toll-like receptor 9	*tlr9*	AY751797	GCCTTCCTTGTCTGCTCTTTCT	GCCGTAGAGGTGCTTCAGTAG
	C-type lectin domain family 10 member A	*clec10a*	KF857329	CGACTCTGGACTCCCTCA	CGTTGTTGATGGTGCGTTC
	Galectin-1	*lgals1*	KF862003	GTGTGAGGAGGTCCGTGATG	ACTGTAGAGCCGTCCGATAGG
	Galectin-8	*lgals8*	KF862004	GGCGGTGAACGGCGGTCA	GCTCCAGCTCCAGTCTGTGTTGATAC
	Fucolectin	*fcl*	KF857331	CCATACTGCTGAACAGACCAACC	TGATGGAGGTGACGATGTAGGA
Mucins	Intestinal mucin	*imuc*	JQ277712	GTGTGACCTCTTCCGTTA	GCAATGACAGCAATGACA
	Mucin 18	*muc18*	JQ277714	ATGGAGGACAGAGTGGAGG	CGACACCTTCAGCCGATG
Apoptosis	Caspase 3	*casp3*	EU722334	GCCAACGGACCTGGACCTG	CCATCGCCTCTCCTCGCATCTA
Antioxidant markers	Catalase	*cat*	JQ308823	TGGTCGAGAACTTGAAGGCTGTC	AGGACGCAGAAATGGCAGAGG
	Superoxide dismutase [Mn]	*sod2*	JQ308833	CCTGACCTGACCTACGACTATGG	AGTGCCTCCTGATATTTCTCCTCTG
	Glutathione S-transferase 3	*gst3*	JQ308828	CCAGATGATCAGTACGTGAAGACCGTC	CTGCTGATGTGAGGAATGTACCGTAAC
	70 kDa heat shock protein, mitochondrial	*hsp70*	DQ524993	TCCGGTGTGGATCTGACCAAAGAC	TGTTTAGGCCCAGAAGCATCCATG
	90 kDa heat shock protein alpha 1	*hsp90α*	KM522802	CTCACAGTTCATCGGCTACCCTATCA	AACTTCCTCTTCCTTCTCTCCCTCATCAAG
Cell growth & regeneration	Proliferating cell nuclear antigen	*pcna*	KF857335	CGTATCTGCCGTGACCTGT	AGAACTTGACTCCGTCCTTGG
	Transforming growth factor beta	*tgfβ*	AF424703	GGAGATTACCGCCTGCTGAC	CCGCTGCTCATCAAGTATCGT
Housekeeping	β actin	*actβ*	X89920	TCCTGCGGAATCCATGAGA	GACGTCGCACTTCATGATGCT
