# Supplementary material for: Acting locally - affecting globally: RNA sequencing of gilthead sea bream with a mild Sparicotyle chrysophrii infection reveals effects on apoptosis, immune and hypoxia related genes
Source: BMC Genomics. 2019 Mar 11;20:200. doi: 10.1186/s12864-019-5581-9 (PMC6416957; doi:10.1186/s12864-019-5581-9)
Supplement: Supplementary file 3 — Pathway classification of DE genes. Bar plots representing the percentage of proteins involved in each broad Reactome pathway. The column labelled Reactome was added as a reference to show the percentage of genes in each category in the database. The other columns represent the percentage of differentially expressed (DE) genes in each category when considering all DE genes in all the tissues of the current experiment (Total) or per tissue, when compared to the control uninfected group. Gill samples are separated in parasitized (P) and non-parasitized (NP) gill sections. (PDF 343 kb) [file 12864_2019_5581_MOESM3_ESM.pdf]

Additional file 3:

Bar plots representing the percentage of proteins involved in each broad Reactome pathway. The column labelled Reactome was added as a reference to show the percentage of genes in each category in the database. The other columns represent the percentage of differentially expressed (DE) genes in each category when considering all DE genes in all the tissues of the current experiment (Total) or per tissue, when compared to the control uninfected group. Gill samples are separated in parasitized (P) and non-parasitized (NP) gill sections.

Pathway	Reactome	Total	Gills_NP	Gills_P	Liver	Spleen
Metabolism	27.41	20.86	20.91	22.63	22.12	18.42
Signal Transduction	15.31	16.87	17.17	15.60	16.59	15.50
Immune System	12.45	13.10	13.99	17.74	14.75	13.74
Gene Expression - Transcription	7.92	8.28	8.59	6.42	7.83	8.04
Developmental Biology	5.89	5.65	5.68	7.03	5.53	5.26
Vesicle-Mediated Transport	4.23	3.99	4.16	3.98	3.69	3.95
Transport of Small Molecules	4.06	5.05	4.99	4.59	4.61	6.14
Hemostasis	4.04	5.72	4.99	5.20	3.23	6.87
Cell Cycle	3.47	2.64	1.80	0.92	3.69	2.63
Cellular Response to External Stimuli	2.70	3.77	3.46	3.06	5.53	4.82
DNA Repair and Replication	2.37	1.73	1.66	0.61	1.84	2.05
Neuronal System	2.07	1.43	1.25	0.61	0.92	1.61
Extracellular Matrix Organization	1.68	2.79	2.63	3.67	1.38	2.34
Organelle Biogenesis and Maintenance	1.66	1.28	1.25	0.61	1.84	1.02
Chromatin Organization	1.35	1.20	0.97	0.31	0.46	1.46
Muscle Contraction	1.15	1.88	2.22	1.83	0.92	1.75
Programmed Cell Death	0.98	1.73	2.08	3.36	2.30	2.05
Cell-Cell Communication	0.73	0.98	0.97	0.92	1.38	0.88
Circadian Clock	0.38	0.98	1.25	0.92	0.92	1.46
Digestion and Absorption	0.15	0.08	0.00	0.00	0.46	0.00
