# Supplementary material for: Acting locally - affecting globally: RNA sequencing of gilthead sea bream with a mild Sparicotyle chrysophrii infection reveals effects on apoptosis, immune and hypoxia related genes
Source: BMC Genomics. 2019 Mar 11;20:200. doi: 10.1186/s12864-019-5581-9 (PMC6416957; doi:10.1186/s12864-019-5581-9)

Additional file 4:

Exploratory analysis of the samples prior to differential expression study. The FPKM density distributions showed three samples with anomalies (red arrows and red in the legend) that were not included in downstream analyses: gill infected parasitized 1 (A), gill infected non-parasitized 5 (B) and spleen infected 2 (D). All liver samples (C) showed uniform distributions and were included in the analyses.

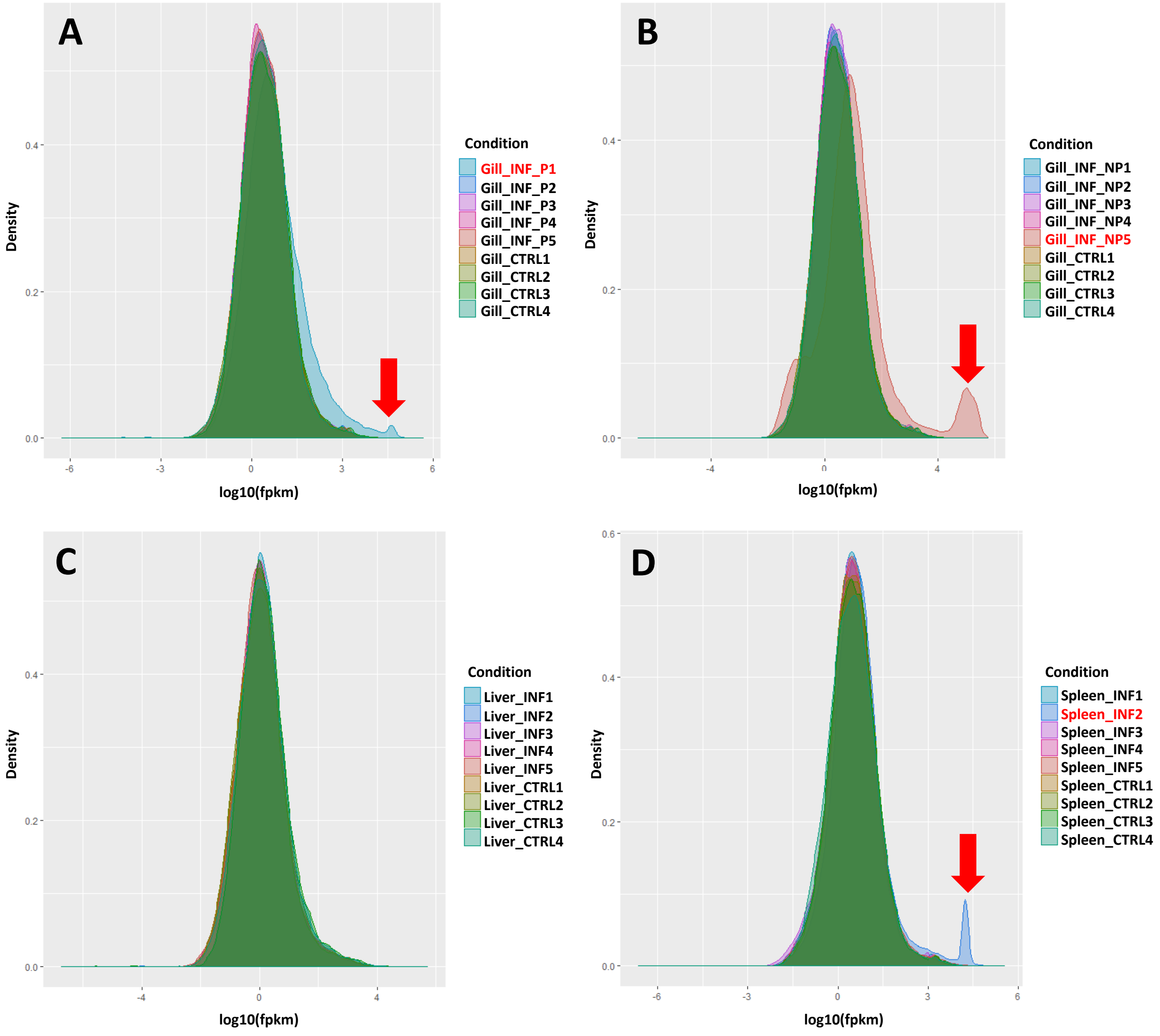

Supplement: Supplementary file 4 — Exploratory analysis of the samples prior to differential expression study. The FPKM density distributions showed three samples with anomalies (red arrows and red in the legend) that were not included in downstream analyses: gill infected parasitized 1 (A), gill infected non-parasitized 5 (B) and spleen infected 2 (D). All liver samples (C) showed uniform distributions and were included in the analyses. (PDF 292 kb) [file 12864_2019_5581_MOESM4_ESM.pdf]
